# Healthcare practitioners’ experiences in managing HIV among young people in Namibia

**DOI:** 10.4102/curationis.v47i2.2608

**Published:** 2024-08-22

**Authors:** Jacques W.N. Kamangu, Sheillah H. Mboweni

**Affiliations:** 1 Department of Health Studies, College of Human Sciences, University of South Africa, Pretoria, South Africa

**Keywords:** experience, healthcare practitioners, HIV viral load suppression, older adolescents, younger adults

## Abstract

**Background:**

Low viral load suppression rates among older adolescents and young adults with HIV are a global challenge, including in Namibia. Healthcare providers struggle with managing these age groups due to their unique demographic characteristics. Monitoring viral load suppression is vital for evaluating antiretroviral treatment effectiveness, making it essential to identify and address existing gaps.

**Objectives:**

This study aimed to explore and describe healthcare practitioners’ understanding and experiences in managing older adolescents and younger adults living with HIV in seven high-burden districts of Namibia.

**Method:**

Qualitative descriptive phenomenological research was followed in this study. Healthcare practitioners directly managing older adolescents and younger adults living with HIV were purposively recruited. Telephonic individual interviews were conducted, and data saturation was achieved with the 29th participant. Colaizzi’s seven-step analysis was used to analyse the data.

**Results:**

Two themes emerged from the study: (1) healthcare practitioners’ knowledge of viral load management and (2) the strategies employed to manage high viral load in these age groups. These strategies included implementing differentiated service delivery, adopting interprofessional and Ubuntu approaches, psychosocial support, community engagement, enhancing adherence counselling, and support from implementing partners.

**Conclusion:**

The findings revealed inadequate knowledge among healthcare practitioners regarding viral load management, which negatively impacts the provision of quality care and an effective HIV response within the spirit of Ubuntu.

**Contribution:**

This study enhances healthcare practitioners’ capacity in viral load management and guides policy makers in supporting this unique population, thus improving their health outcomes.

## Introduction

With advancements in antiretroviral therapy (ART), people living with HIV are now able to live longer, resulting in a growing population of older adolescents and younger adults with HIV (Aderemi-Williams et al. 2021). However, managing the healthcare needs of this specific age group presents unique challenges that require specialised knowledge and approaches. This research article aimed to explore the knowledge and experiences of healthcare providers (HCPs) in providing care and support to older adolescents living with HIV (OALHIV) and younger adults living with HIV (YALHIV). At the end of 2022, the World Health Organization (WHO) approximated that globally 30.9 million people of all ages were living with HIV, with 86% aware of their HIV status, 76% receiving ART medication and 71% achieving viral load suppression (VLS). However, the VLS rate of 71% falls significantly short of the Joint United Nations Programme on HIV/AIDS (UNAIDS) (2023) target of 95-95-95 by 2030.

Out of 30.9 million people living with HIV, 5 million are young people aged 15–25 years, and 1.5 million are children aged 0–14 years, which raises grave concerns (WHO 2023a). Older adolescence and young adulthood is a crucial stage of sexual, physical and emotional development, and it is during this stage that most acquire HIV through sexual activity (UNAIDS 2023). Two out of every seven new HIV infections worldwide are among young people aged 15–24 years (UNAIDS 2019). Moreover, UNAIDS (2023) reports that 53% of those living with HIV are women and girls aged 15–24 years. Additionally, UNAIDS (2023) is concerned that Africa remains the region of impact with 20.9 million people living with HIV. The global AIDS strategy for 2021–2026, aimed at ending inequalities and AIDS, seeks to empower young individuals, particularly adolescent girls and young women aged 15–24 years, by allocating resources to HIV response initiatives tailored to this demographic, to reduce new infections (UNAIDS 2021a).

The world is still behind in achieving the targets set for these age groups as is evident in the low VLS rate, and therefore, a holistic targeted intervention is required as these individuals are at risk of developing drug resistance and virologic failure (UNAIDS 2021b). Additionally, according to the UNAIDS report from 2023, despite a 59% decrease in new HIV infections since 1995 and a 69% decrease in AIDS-related deaths since 2010, substantial numbers of new HIV infections persist within these age groups. Particularly concerning is the significant number of new HIV infections among adolescent girls and young women, who represented 46% of all new infections globally in 2022. Moreover, the sub-Saharan region accounted for over 77% of new infections among individuals aged 15–24 years in the same year (UNAIDS 2023).

Adolescents and younger adults acquire HIV because of their heterogeneous nature, exacerbated by their developmental stage, which encompasses various factors such as physical, cognitive, communication, language, social, emotional and emerging sexual identity issues (Bekker et al. 2015). These factors significantly influence their maturity levels, treatment adherence and willingness to disclose their HIV status. Consequently, it is vital to meticulously consider these distinctive characteristics, as they can potentially undermine HIV treatment outcomes and complicate clinical decision-making processes (Clinical Info HIV 2024).

Adherence is key to achieving VLS among adolescents and younger adults. A study conducted by Kamangu and Mboweni (2024) in Namibia revealed various factors that can affect the management of OALHIV and YALHIV; these include patient, health facility, community and HCP factors, and should be taken into consideration when managing these age groups. Healthcare providers’ knowledge and experience in managing these age groups is key and requires interprofessional collaborative practice, care and management of people living with HIV to ensure quality care (WHO 2010). Interprofessional collaboration involves the collaboration of HCPs with diverse backgrounds, including nurses, doctors, community health workers, physiotherapists, social workers, counsellors, and pharmacists working together alongside patients, families, caregivers, and communities to provide the utmost level of care (Open Resources for Nursing, Ernstmeyer & Christman 2022; WHO 2010; Woollett, Pahad & Black 2021). This also signifies the application of Ubuntu, our African philosophy, in providing care for people living with HIV. This philosophy or concept is rooted in African culture, and encompasses the values of humanity, community, sharing, and care. It emphasises the significance of cooperation and solidarity among individuals, cultures and nations. The ethical principles of Ubuntu include respect for others, willingness to help, fostering a sense of community, promoting sharing and caring, trust, and selflessness. Ubuntu forms the foundation for fostering community spirit, nurturing familial bonds, ensuring fairness in social justice, and cultivating a sense of unity among diverse groups of people (Nicolaides 2023; Nzimakwe 2014). Exploring Ubuntu can be instrumental in combating epidemics, informing decision-making processes, and integrating it into HIV training programmes (Sambala, Cooper & Manderson 2020). Therefore, HCPs and public health leaders seeking to enhance the delivery of quality HIV services should delve further into the ethical principles of Ubuntu.

Therefore, the role of HCPs in managing OALHIV and YALHIV is critical, and exploring their experiences can help improve the care and management of these age groups. Each HCP should play a specific role in providing comprehensive care. In addition to fulfilling their responsibilities, HCPs need continuous training, education, and professional development to remain abreast of the latest advancements in research, innovative technologies, treatment methods, guidelines and evidence-based practices. This includes adapting to regulatory alterations to improve clinical competency, support personal and professional advancement, and provide patient-centred care (Mlambo, Silén & McGrath 2021). Even though Namibia seems to be doing well when combining all age groups at 95-97-94 of the UNAIDS target, it is not doing well among OALHIV and YALHIV and this calls for attention (Ministry of Health and Social Services 2023). Namibia has VLS of 63% of adolescents 0–14 years of age and 60.5% of OALHIV and YALHIV 15-24 years of age, which is well below the national and WHO average suppression levels, despite teen clubs being introduced in 2010. There are still barriers and gaps in current practice faced by HCPs, and these gaps should be investigated (Munyayi & Van Wyk 2020) and dealt with decisively at all levels of care. These challenges and gaps include quality issues, human resource shortages, insufficient budget allocations, ineffective leadership and management, limited access to telemedicine and digital healthcare, absence of age-specific programmes, guidelines and policies, a lack of political commitment, and inadequate training and capacity-building among HCPs, patients and caregivers. These challenges can compromise health outcomes significantly, particularly within the public health sector (Maphumulo & Bhengu 2019; Mboweni 2024; Oleribe et al. 2019).

Namibia also faces similar challenges of poor adherence and low VLS among OALHIV and YALHIV. Limited studies are exploring the management and care of OALHIV and YALHIV in the country compared to adults living with HIV, and more studies should be conducted to provide quality targeted, and comprehensive intervention for this age group. It was therefore important to conduct a study and explore HCPs’ knowledge and experiences regarding the management of OALHIV and YALHIV to improve adherence, VLS, and the overall health outcomes of this age group.

## Research methods and design

### Study design

A qualitative descriptive phenomenological research approach was employed to explore and gain an in-depth insight into the experiences and perspectives of HCPs within the context of Namibia high burden districts (Dawadi, Shrestha & Giri 2021). This study design is one of the most commonly used methodologies in qualitative research within the social and health sciences; it is employed to describe the essence of human beings’ experiences as they are lived and perceived by the individuals in a certain phenomenon.

### Population and sampling

The population comprises all individuals, organisations, groups, or entities of interest that one wants to understand and to which the research results may be transferred and generalised (Casteel & Bridier 2021; Majid 2018). The population of the study included all HCPs managing people living with HIV; these HCPs included registered and enrolled nurses, medical doctors and health assistants. Participants were recruited using a non-probability purposive method from the seven high-burden healthcare facilities providing HIV care and treatment services for at least 12 months without interruption. This allowed the researchers to select participants who possess the specific experience regarding managing OALHIV and YALHIV to access their specialised knowledge and expertise, thus gaining valuable insights and understanding about the phenomenon (Creswell & Creswell 2018).

### Study setting

The study took place in six regions of Namibia: Khomas, Ohangwena, Omusati, Oshana, Oshikoto and Zambezi. There was one high-burden district in each region except in Omusati, where two high-burden districts were identified. These regions were specifically selected because of their high-HIV and AIDS-burden districts with low VLS rates among OALHIV and YALHIV. Together, these six regions represented 80% of the target population and accounted for the majority of people living with HIV in Namibia. These high-burden districts include Windhoek (15.5%), Engela (15.9%), Outapi (8.7%), Oshikuku (11.8%), Oshakati (15.2%), Onandjokwe (16.0%), and Katima (16.9%). The focus of the study was primarily on the northern part and peri-urban areas, characterised by poorer socio-economic conditions, in contrast with the central part of the country, represented by the Khomas region, which is fully urban area. The Ministry of Health and Social Services (MoHSS), specifically the Directorate of Special Programmes (DSP) in HIV and Sexually Transmitted Infections (STI), leads the HIV response and control programme in Namibia, and oversees the efforts to combat the HIV epidemic (that is where the principal researcher holds a position as Deputy HIV Chief Clinical Mentor).

#### Data collection

Semi-structured individual telephone interviews were conducted by the primary researcher to gather rich and detailed information about the phenomenon (Brink, Van der Walt & Van Rensburg 2018). The research received approval in November 2021 amid the coronavirus disease 2019 (COVID-19) pandemic. To adhere to infection control policies and restrictions, telephone interviews were chosen as the most suitable method to engage with HCPs from the designated high burden districts. Data were collected in English, as all HCPs were conversant with this language, and the interviews were audio recorded with the permission of the participants. Initially, a comprehensive list of 84 HCPs responsible for HIV management in seven districts with high HIV burden was compiled. This list, along with the contact information, was obtained through the assistance of facility managers who served as gatekeepers. The gatekeepers informed the HCPs about the research during their staff meetings. Healthcare providers who expressed interest in participating provided their names to the gatekeepers, who then shared this information with the principal researcher. The principal researcher used this information to arrange interview appointments. Two months prior to data collection, the HCPs were provided with an information leaflet containing detailed study information, including the inclusion criteria and consent forms through their facility email. Sufficient time was given to the participants to review the materials, complete the consent forms and schedule the interviews.

Those interested responded by sending back the signed consent form, and appointments were made with them at a time convenient for them during lunch or when they were off duty to avoid interruptions in service delivery. The principal researcher secured a private space for himself and initiated the telephonic interviews, which lasted approximately 45 min to an hour. Participants were able to select their own private space, as described in the leaflet, to ensure uninterrupted interview environment. Participants did not bear any airtime costs during the interviews; calls were initiated by the researcher. Data were collected between January and May 2022. A total of 53 HCPs agreed to participate, but only 29 ultimately participated in the study, and data saturation was reached with this sample. Data saturation occurs when no new information or themes emerge during an interview, or when themes begin to repeat, indicating that the data collection process has reached a point of redundancy and further interviews are unlikely to yield novel insights (Hennink, Kaiser & Weber 2019; Saunders et al. 2018). Others declined and some did not take the researcher’s calls.

Despite the absence of direct visual access to participants during telephone interviews, the researcher employed techniques to capture non-verbal reactions and emotions. This was achieved through active listening, attentively noting the participants’ tone, pitch and pace, which provided insights into their emotional states and engagement levels. The researcher also paid attention to pauses and hesitations during the interview, utilising probing skills to gather additional information about these nonverbal cues (Azad et al. 2021). Field notes were taken to document these observations. The audiotapes were kept separately from the consent forms, the list of participants and transcribed data so that no one can link the three documents. Furthermore, transcribed data sheets were labelled using codes instead of the names of participants to ensure confidentiality and anonymity. The audiotapes and transcribed data files were stored separately on a password-protected computer system, ensuring that only the researcher has access to them.

#### Data analysis

Data were analysed using computer-assisted qualitative data analysis software (CAQDAS) supported by manual data analysis following Colaizzi’s seven steps of data analysis. A software system cannot support automatic theming and cannot reduce bias or increase reliability and validity on its own (Praveena & Sasikumar 2021; Colaizzi 1978).

Step 1 involved transcribing audio-recorded interviews verbatim, followed by the researcher reading and re-reading the written script, and at the same time, listening to the audio again to gain a sense of the whole document. Step 2 involved extracting significant statements and phrases about the management of OALHIV and YALHIV, and formulating meanings as step 3. These were reviewed and discussed by the two researchers until a consensus was reached. In step 4, the formulated meanings were organised into clusters of themes, and these became the emergent themes of the study and were presented to the researcher experienced in qualitative studies to check for accuracy. In step 5, all emergent themes were described comprehensively and scrutinised by an experienced researcher to assess their richness and completeness to ensure exhaustiveness concerning strategies used to manage OALHIV and YALHIV. Step 6 involved describing the fundamental structure of the participants’ descriptions regarding the exploration of experiences of HCPs regarding the management of these age groups. Lastly, the researchers returned to the participants to validate the findings through member checking techniques by means of telephone interviews so that the participants could confirm if the findings reflected their experiences and feelings.

### Measures of trustworthiness

Ensuring trustworthiness is essential in qualitative research to mitigate potential bias and establish the credibility, reliability, dependability, confirmability, and transferability of the findings (Haradhan 2018; Korstjens & Moser 2018). Different strategies were applied to ensure trustworthiness. Credibility was enhanced through extensive engagement and interaction with participants, fostering a comprehensive comprehension of their context and experiences. The interviews had a duration of approximately 45 min to 1 h. Additionally, the recorded interviews and transcriptions underwent a thorough review by a second researcher, who identified any discrepancies that were subsequently discussed and resolved. The data processing and derived themes from the study findings were also subjected to critical analysis by an independent qualitative research expert to ensure credibility. Transferability was reinforced by offering detailed and comprehensive descriptions of the research context and the data collection process. The study findings were supported by participants’ quotes, which illustrated the key themes and perspectives described in the results sections. This enabled researchers and readers to evaluate the applicability of the findings to analogous situations. Dependability was ensured through thorough documentation of the research processes, encompassing data collection, analysis and interpretation. These processes were explained to enable the reader to better understand the study flow. Additionally, the first researcher presented the findings to the second researcher for validation, further reinforcing dependability. Confirmability was attained through member checking, where nine participants were invited to review and offer feedback on the findings, ensuring accuracy and mitigating researcher bias (Forero et al. 2018).

### Ethical considerations

The research study received ethical approval from two entities: the University of South Africa Research Ethics Committee (reference number 12786918_CREC_CHS_2021) and the Ministry of Health and Social Services Research Ethics Committee (reference number: 22/4/2/3), from which permission to conduct the study was obtained. The study followed ethical principles in accordance with the Helsinki Declaration, which guides the ethical conduct of research involving human participants, by obtaining written informed consent from participants and ensuring their autonomy by voluntarily asking them to take part in the study without any coercion. Furthermore, the researchers ensured compliance with the ethical principles of non-maleficence by addressing any potential discomfort associated with the interviews. This was accomplished through displaying empathy, sensitivity, and respect towards participants’ emotions and experiences. Measures such as assuring confidentiality and conducting debriefing sessions at the conclusion of the interviews were implemented to provide reassurance and support to the participants (Varkey 2021). To ensure beneficence, the study purpose was explained to the HCPs, emphasising its significant contribution to enhancing the overall knowledge and understanding of healthcare practices and in managing OALHIV and YALHIV. This was achieved by providing detailed information about the study to participants through information leaflets and addressing all questions and concerns to ensure understanding, voluntary participation and non-maleficence.

Confidentiality and anonymity were reinforced by employing codes rather than names during the interview records. Participants were treated with respect and dignity, and they were afforded the right to withdraw from the study at any stage without facing any penalties (Creswell & Creswell 2018; Polit & Beck 2021). All participants were treated equally and fairly throughout the entire process, were selected purposively, and had rights to withdraw at any time without any penalties.

## Results

### Characteristics of study participants

The study involved a total of 29 participants. Their years of experience in managing people living with HIV ranged from 1 to 16 years, with a median experience of 5.3 years. The study findings indicate that participants had significant experience in managing HIV. The majority of the participants were nurses, comprising 68.9% of the total participants. Among them, 55.0% were registered nurses and 13.7% were enrolled nurses. It is worth noting that the main workforce of the MoHSS consists of nurses who are trained in HIV management. The study also included three medical doctors (10.3%) and six health assistants (20.6%). The gender distribution among participants showed that more females (80%) than males (20%) participated in the study, consistent with the composition of the Namibian health workforce, as outlined in Table 1.

**TABLE 1:** Summary of participant demographics.

No.	Participant ID number	Gender	Position	Years of experience
1.	P01RN	Female	Registered nurse	4
2.	P02RN	Female	Registered nurse	3
3.	P03HA	Female	Health assistant	15
4.	P04HA	Male	Health assistant	4
5.	P05EN	Female	Enrolled nurse	6
6.	P06RN	Female	Registered nurse	10
7.	P07EN	Female	Enrolled nurse	6
8.	P08EN	Female	Enrolled nurse	16
9.	P09RN	Female	Registered nurse	6
10.	P10RN	Male	Registered nurse	8
11.	P11HA	Female	Health assistant	7
12.	P12EN	Female	Enrolled nurse	6
13.	P13RN	Female	Registered nurse	5
14.	P14RN	Female	Registered nurse	3
15.	P15RN	Male	Registered nurse	5
16.	P16RN	Female	Registered nurse	1
17.	P17RN	Female	Registered nurse	5
18.	P18HA	Female	Health assistant	12
19.	P19RN	Female	Registered nurse	9
20.	P20RN	Male	Registered nurse	5
21.	P21RN	Male	Registered nurse	3
22.	P22HA	Female	Health assistant	3
23.	P23RN	Female	Registered nurse	3
24.	P24HA	Male	Health assistant	8
25.	P25RN	Female	Registered nurse	4
26.	P26RN	Female	Registered nurse	3
27.	P27DR	Female	Medical doctor	7
28.	P28DR	Female	Medical doctor	2
29.	P29DR	Female	Medical doctor	3

### Study results

The study reveals two major themes related to HCPs’ knowledge and experience in managing OALHIV and YALHIV, with subthemes summarised in Table 2. These themes were firstly, knowledge gaps related to viral load management and secondly, current strategies on the management of OALHIV and YALHIV with high viral load (HVL) by HCPs to achieve the desired level of VLS.

**TABLE 2:** Themes and subthemes regarding healthcare providers’ experiences in managing older adolescents living with HIV and younger adults living with HIV.

Themes	Subthemes	Categories
Knowledge gap related to viral load monitoring	Inadequate description of viral load	-
	Inadequate description of treatment failure	-
	Inadequate description of facility VLS rate	-
HCPs’ experiences regarding strategies to manage high viral load among OALHIV and YALHIV	Differentiated service delivery approach	Viraemia clinic day
		Direct ART observed therapy
	Psychosocial support	Teen clubs
		Namibian Adolescents Treatment Supporters
	Community involvement	Community-based organisation involvement
		School involvement
		Guardians or caregiver involvement
	Interprofessional and Ubuntu approaches	-
	Enhanced adherence counselling	-
	Implementing partner support	Contact tracing and home visits
		Incentive to support adherence

HCP, healthcare provider; OALHIV, older adolescents living with HIV; YALHIV, younger adults living with HIV; VLS, viral load suppression; ART, antiretroviral therapy.

### Theme 1: Knowledge gap related to viral load monitoring

Through the individual interviews, the researchers evaluated the extent of participants’ understanding of viral load monitoring, and the findings point out several areas of concern. These are insufficient explanations of viral load, insufficient explanations of treatment failure and a low rate of VLS among OALHIV and YALHIV in their respective facilities. These findings raise concerns as they could potentially hinder efforts to decrease new HIV infections and hinder the goal of ending the HIV or AIDS epidemic by 2030.

#### Inadequate description of viral load

During the interviews, not all participants were able to provide sufficient explanations of what viral load entails. However, the majority demonstrated a good understanding of viral load, although they faced challenges in describing VLS and viral load tests. This was not expected based on their years of experience, that is, 12 months and more, of managing people living with HIV in those facilities. The following responses were recorded from participants:

‘HIV viral load refers to the quantity of the virus present per millilitre of blood in a person’s body, measured per copy in a million.’ (P01RN)

‘Viral load suppression is achieved when an individual living with HIV has reached a minimum level, as outlined in our guidelines, which is typically less than 40 copies. So, when a client’s viral load is below 40, we consider them to be virally suppressed.’ (P09RN)

‘VL testing is crucial because it helps us assess the effectiveness of the medication we administer, ensuring that clients are adhering to their prescribed treatment and not missing any doses.’ (P17RN)

There were challenges in describing VLS and viral load tests accurately, and some participants struggled to explain viral load. Some of them displayed an inadequate understanding:

‘HIV viral load refers to a higher number of the virus within someone’s body. This could be because of poor medication adherence or delayed HIV testing, resulting in an elevated viral load.’ (P03HA)

‘Viral load suppression occurs when the amount of viral load in the body of an individual living with HIV is low, indicating successful medication intake.’ (P02RN)

#### Inadequate description of treatment failure

The majority of participants demonstrated a limited understanding of treatment failure. They failed to describe the three stages of treatment failure, namely virologic, immunologic and clinical failure. Many participants focussed primarily on the initial stage of virologic failure, with some neglecting to mention any of the stages altogether. This raises significant concerns, as understanding all stages of treatment failure is crucial for preventing disease progression and the development of ART resistance. The following participant quotations support these observations:

‘Treatment failure encompasses immunology failure and clinical failure. It occurs when a patient experiences persistent symptom despite adhering to treatment, and their condition progresses to stage four or three. Treatment failure is categorized into stages, assessing the patient’s lack of progress despite being on treatment and the inability to suppress the virus. It involves clinical evaluation and other factors.’ (P28DR)

‘Treatment failure is when a patient consistently has a high viral load that remains unchanged for an extended period. This can sometimes be due to the patient not adhering to the correct medication regimen.’ (P07EN)

‘Treatment failure occurs when a patient fails to take their medication as prescribed or adhere to the clinical schedule. This can occur over time, such as when a patient exceeds the recommended interval of 14 days without taking the medication. In some cases, the patient may completely disengage from treatment and fail to visit other clinics despite being due for follow-up.’ (P04HA)

#### Inadequate description of facility viral load suppression rate

While some participants were aware of the VLS rate at their facilities, most did not know their facility’s VLS rate. Those who knew reported low VLS rates. This situation negatively impacts the goal of reducing new HIV infections, especially in the absence of safer sex practices, and underscores the need for additional efforts to achieve optimal VLS. One of the participants expressed with confidence the following perspective on both good and low VLS rates:

‘At my clinic, the rate is 100% because all older adolescents and young adults living with HIV have viral loads below 40 copies.’ (P16RN)

Some expressed concern about the current VLS rate:

‘No, it is still not enough because we still have that 10% who are not suppressed. This is not satisfactory. Our target is to achieve HIV-free status in Namibia by 2030, but with high viral loads persisting, we will struggle to reach that goal.’ (P01RN)

Some participants were unable to provide exact calculations of the VLS rate within their facilities. Instead, they shared the total number of OALHIV and YALHIV who had unsuppressed viral loads or provided estimated figures:

‘I am aware of the number of individuals with suppressed viral loads, but I do not have the exact count for those who are not yet suppressed. However, among older adolescents [*aged 15–19*], there are five individuals, and among young adults [*aged 20–24*], there are nine individuals with high viral loads.’ (P21RN)

‘I cannot provide an exact figure, but our observations indicate that we do not have many individuals with high viral loads. However, we do have a few individuals with low levels of viremia.’ (P09RN)

### Theme 2: Healthcare providers’ experiences regarding strategies to manage high viral load among older adolescents living with HIV and younger adults living with HIV

#### Differentiated service delivery approach

Participants shared that they employed a patient-centred approach to address the issues faced by some OALHIV and YALHIV who have unsuppressed viral load or adherence problems. This approach included the implementation of a viraemia clinic day and directly administered antiretroviral therapy (DAART). The differentiated service delivery approach refers to a framework or strategy that aims to provide healthcare services tailored to the specific needs and circumstances of individuals or groups. It recognises that not all patients require the same level or type of care and seeks to optimise resources by offering differentiated services based on factors such as clinical condition, treatment stage, geographical location and patient preferences. Furthermore, differentiated service delivery recognises that a one-size-fits-all approach may not be suitable for all patients and seeks to provide individualised care that optimises health outcomes while considering factors such as patient preferences, convenience and the efficient use of healthcare resources.

**Viraemia clinic day:** On a designated viraemia clinic day, healthcare professionals concentrate on providing targeted care, such as enhanced adherence counselling and referrals to dedicated multidisciplinary team interventions. Additionally, this facilitates improved documentation in the tools used to monitor patients with HVLs. The following participant statements support this approach:

‘We reserve Fridays exclusively for these individuals. It is our designated high viral load day for both adolescents and adults. On this day, those with high viral loads come in, and we provide enhanced adherence counselling. We also refer them to the Namibia Adolescents Treatment Support.’ (P01RN)

‘These individuals are scheduled to be seen on a chosen day, either once a week or once a month, to allow for focused attention during their clinic visit. The clinic enhanced documentation and improved continuity of care.’ (P05EN)

**Direct ART observed therapy:** Participants highlighted the importance of the implementation of DAART, adapted from the directly observed therapy approach used for tuberculosis management. It is employed for patients with adherence challenges who reside in close proximity to the health facility. For patients living further away, the administration of ART is overseen by a treatment supporter or community health worker. This approach ensures daily medication intake and allows HCPs to confidently conduct repeat viral load tests, contributing to improved management strategies.

The participants described the situation as follows:

‘If a client’s viral load is high, we monitor them by conducting viral load tests every three months and providing adherence counselling during their visits. As a facility, we also discuss ways to support these clients. If the client lives nearby, we use a DOT [*directly observed therapy*] system, where they come to the clinic to collect their medication and take it under close supervision. This allows us to closely monitor whether the client is taking their medication.’ (P11HA)

‘The role of the facility is to communicate to the parents, carer and do counselling. Usually, if a patient cannot take the medication by her/himself, we do DAART, Sometimes we do get help of parent or carers. This one is just at home.’ (P08EN)

#### Psychosocial support

Participants highlighted the provision of psychosocial support to OALHIV and YALHIV with HVLs through teen clubs or referral to peer-led support programmes such as Namibian Adolescents Treatment Supporters (NATS). Teen clubs serve as platforms for adolescents living with HIV, aiming to provide psychosocial support and foster confidence in medication adherence. Participants emphasised the importance of peer learning and support through these clubs in promoting positive living and achieving VLS.

**Teen clubs:** Participants indicated that they utilised teen clubs as platforms for adolescents aged 10 to 19 years living with HIV, irrespective of their viral load status. The teen clubs aim to deliver psychosocial support and foster confidence among adolescents living with HIV, encouraging them to accept and adhere to their treatment. Similarly, some facilities employ young adult clubs to instil confidence in YALHIV. The participants described this as follows:

‘At our facility, we have a teen club where young adolescents gather, and a dedicated healthcare worker attends with them. During these sessions, the healthcare worker imparts health-related knowledge in the context of HIV. The teen club is not limited to those with high viral loads; it includes all adolescents. By teaching each other how to live positively, they learn the importance of adhering to medication and living a fulfilling life like their peers, ultimately leading to viral load suppression. This is how we manage them.’ (P06RN)

‘We have a teen club that meets every month, and we also call some individuals for discussions about why their viral load is high. We provide health education both in groups, like the teen club and individually. At our facility, we see school-going teens from 14:00, which makes it easier to talk to them.’ (P12EN)

**Namibian Adolescents Treatment Supporters:** Participants highlighted the role of NATS in providing peer-led support for OALHIV and YALHIV with HVLs. These trained YALHIV, who have achieved VLS, offer psychosocial support and counselling to their peers in a language they understand. Namibian Adolescents Treatment Supporters maintains records, conducts follow-ups and actively reaches out to patients, ensuring their engagement in care. The presence of NATS provides OALHIV and YALHIV with a sense of hope and solidarity, knowing that they are not alone in their treatment journey. The participants had the following to say:

‘We have the NATS, and if I recall correctly, they are sponsored by Potentia. They maintain a record book where they document all the patients with high viral loads, their follow-ups, and the scheduled dates for their next follow-up. They actively reach out to these patients, call them, and track them down to ensure they come to the clinic. They engage in counselling sessions with them because they, too, are on antiretroviral therapy just like the adolescents.’ (P07EN)

‘NATS are available in our clinic who ensure that children and young PLHIV aged 0 to 19 and 20 to 24 have good adherence. When they come to the clinic, we see them first. We also set aside a day for counselling individuals aged 0 to 24, which we conduct in a friendly manner. This has helped us significantly in suppressing the viral load.’ (P15RN)

#### Community involvement

**Community-based organisation involvement:** Participants indicated that the community plays a significant role in the management of OALHIV and YALHIV, irrespective of their viral load status. Since these age groups are not fully independent, they rely on their parents, guardians, or supervisors for support. The participants emphasised the involvement of guardians, community-based organisations and schools in the management of OALHIV and YALHIV, particularly in ensuring adherence to ART medication in the spirit of Ubuntu to eradicate stigma and discrimination. Rural communities embrace the ethos of Ubuntu and can serve as a foundation for community-led support initiatives.

**Guardian or caregiver involvement:** Some participants mentioned the involvement of guardians in the management plan for OALHIV and YALHIV who have HVLs. Healthcare providers define guardians as biological parents, adult family members, or individuals within the community who have been entrusted with the responsibility of caring for OALHIV and YALHIV. The guardians play a crucial role in collaborating with HCPs, providing support and supervising the administration of medication to OALHIV and YALHIV, as well as ensuring routine follow-up. Their involvement is vital in complementing the efforts of HCPs and monitoring the well-being of OALHIV and YALHIV. The participants said:

‘As HCPs, we establish a system that brings together the children, their parents, and other willing treatment supporters. We alone cannot accomplish everything because we only see these children once a month, but a parent or guardian can closely observe their behaviours and academic performance, which can assist us in making improvements.’ (P04HA)

‘When we detect a high viral load, we provide enhanced adherence counselling. We also call in the guardian to ask why the child is having a problem. We must rule out all other factors that might contribute to the issue. We then prescribe one month of counselling. Whenever they come together with the guardian for follow-up, we provide adherence counselling and conduct a pill count to determine how they are taking their medication.’ (P25RN)

**School involvement:** Participants highlighted the involvement of schools in the care of OALHIV and YALHIV. In some schools, efforts are made to synchronise clinic visits for medication refills with their school schedules. Schools with designated Life Skills teachers provide guidance and support to OALHIV and YALHIV in adhering to their ART medications. However, this support is contingent upon the disclosure of their HIV status to the Life Skills teacher with parental or guardian consent. Collaboration among parents or guardians, OALHIV, YALHIV and schools, particularly in boarding school settings, has played a pivotal role in promoting adherence among these individuals:

‘At our facility, we schedule appointments for school-going individuals from 2 p.m., which makes it easier to communicate with them. They are aware of who is taking medication, and we suggest that they all come together for a brief discussion before returning to class. This is how we manage their care.’ (P12EN)

‘We work with life skill teachers from local schools and boarding schools and train them on how to support and monitor adherence with parental consent.’ (P28DR)

#### Interprofessional and Ubuntu approaches

Participants highlighted the HCPs coming together as a multidisciplinary team to address adherence and unsuppressed viral load issues among OALHIV and YALHIV. However, they noted that many individuals in these populations prefer individual interactions with specific HCPs, particularly social workers, doctors and nurses. This preference for individual interactions raises concerns that need to be considered when developing HIV programmes to enhance adherence through the values of Ubuntu. The involvement of social workers and home visits were mentioned as important strategies to address these concerns:

‘When we identify an adolescent with a high viral load at our facility, we usually schedule them for a specific day, which is Thursday, to provide comprehensive counselling. We involve various team members, including National Adolescent Treatment Supporters [*NATS*], health assistants, regional clinical mentors, and nurse mentors, to discuss the way forward. If there are any issues, we engage the social worker to conduct home visits.’ (P19RN)

‘After the counselling, the doctor prescribes the necessary medication. On certain occasions, we also use a multidisciplinary approach involving two nurses, the doctor, the health assistant, the NATS, and sometimes a social worker. We conduct a session with the patient and encourage them to be open.’ (P23RN)

#### Enhanced adherence counselling

The majority of participants indicated their colleagues at the facility implement enhanced adherence counselling (EAC) as a means to improve adherence among people living with HIV, including OALHIV and YALHIV. Although EAC is a standardised intervention recommended in the national ART guidelines, it is not always sufficient to address every case of poor adherence and detect HVL alone. Therefore, this intervention should be complemented with other strategies such as *Ubuntu principles* to increase the likelihood of effectively addressing adherence issues. Participants provided supporting evidence for this statement:

‘We have a teen-club and youth club at the facility and they are divided amongst us and the NATS to be given health education and do continuous adherence counselling every month when they come, and viral load after three months.’ (P19RN)

‘For those with a high viral load, we pull and flag their files for close monitoring. When they return, they must go for adherence counselling to understand why they have a high viral load and manage their treatment based on the reasons they provide.’ (P09RN)

#### Implementing partner support

Participants highlighted that various implementing partners play a supportive role in the HIV response in Namibia, particularly at the community level. The implementing partners referred to in this context are non-profit organisations (NPOs) and non-governmental organisations (NGOs) that receive funding from the President’s Emergency Plan for AIDS Relief (PEPFAR). These partners are dedicated to combating the HIV epidemic and working towards achieving the goal of ending it by 2030. Implementing partners, such as Project HOPE, DREAMS and the Total Control of the Epidemic (TCE)/Development Aid from People-to-People programme, collaborate with HCPs at healthcare facilities to enhance adherence among people living with HIV through their community-based workforce. These partners employ community health workers for tasks such as contact tracing and home visits. In addition, they provide incentives to promote adherence among OALHIV and YALHIV.

**Contact tracing and home visits:** Participants highlighted the valuable role of community health workers in contact tracing and home visits for OALHIV and YALHIV. These workers diligently track down individuals who have missed their clinic appointments or medication refills, ensuring that they are brought back into care. Home visits are conducted to provide supportive services and counselling to those facing adherence challenges. Total Control of the Epidemic/Development Aid from People to People, as an implementing partner, employs a TRIO (Tool for Risk Interventions and Outcomes) model to further support adherence, involving the patient, field officer and caregiver. Project HOPE also contributes to patient tracing efforts. Participants provided the following details to support this observation:

‘TCE is also assisting us. They help us in locating the patients. If a patient has interrupted their treatment and their contact number is unreachable, TCE helps us by conducting field visits to locate the patient. Additionally, we have Project HOPE, which also supports us in tracing the patients.’ (P05EN)

‘We have other stakeholders, such as IntraHealth and TCE. These two organizations help track patients because they frequently work in the community.’ (P04HA)

**Incentive to support adherence:** Participants reported that in addition to contact tracing and home visits, implementing partners such as Project HOPE and DREAMS offer incentives to support adherence among OALHIV and YALHIV. DREAMS (Determined, Resilient, Empowered, AIDS-free, Mentored, and Safe) is an initiative focussed on reducing new HIV infections among adolescent girls and young women in sub-Saharan Africa. It aims to address the structural factors that contribute to HIV vulnerability among this population, such as poverty, gender inequality, lack of education and limited access to healthcare. These incentives aim to encourage adherence to ART and overall well-being among the target population. Healthcare providers expressed concerns about the long-term sustainability of the contracts with these partners, emphasising the need for the government to take action instead of relying solely on external partners. Participants provided the following details to support this statement:

‘We have Project HOPE and TCE. Unfortunately, the contract with Project HOPE has ended, but they helped us with our adolescents. They provided healthcare workers who were assigned to our adolescents, closely monitoring them by accompanying them to their homes, conducting adherence counselling with caregivers and parents, and assisting with birth certificate registrations if needed.’ (P09RN)

‘Project HOPE even provided food for the children. They went house to house to deliver the food and used to give transport money for the teen club.’ (P03HA)

## Discussion

The findings highlight the importance of targeted training programmes, educational and continuous professional development initiatives to enhance HCPs’ knowledge and understanding of the specific healthcare needs of older adolescents and younger adults living with HIV, more specifically viral load monitoring. Strategies to improve management, adherence, disclosure, counselling and communication, including building trust between HCPs, patients, family and caregivers in these age groups, are also crucial. Additionally, addressing the barriers to accessing appropriate healthcare services, such as patient literacy, stigma and discrimination, is vital to ensure comprehensive care.

### Knowledge related to viral load monitoring of older adolescents living with HIV and younger adults living with HIV

The study findings reveal that most HCPs are well versed in the definition of viral load as the amount of the virus in the blood. The HCPs’ definition of viral load is in line with that of UNAIDS which describes HIV viral load as the number of HIV viral particles per millilitre of blood and the monitoring approach is preferred to detect and confirm the failure of ART (UNAIDS 2020; WHO 2019b). However, the results highlight that HCPs know little about the treatment failure and their facility’s VLS rate. Poor knowledge of HIV and treatment failure are associated with poor health education, information, communication and counselling skills for beneficiaries such as adolescents and younger adults. A study conducted by Mboweni and Makhado (2020, 2022) in South Africa also found that even nurses trained in nurse-initiated management of ART lack the skills and competence to provide quality care as they struggle to implement what they have been trained, especially the management of children and adolescents, and require mentoring, support and ongoing in-service training.

This was also revealed by a study conducted by Iseselo et al. (2024) in Tanzania on the challenges experienced by HCPs in the delivery of health services for people living with HIV in Dar es Salaam. This study raised a concern that the lack of up-to-date knowledge and limited access to health information among HCPs limit the provision of high-quality care to individuals living with HIV. This is worrisome as the prevalence of HIV is high among adolescent girls and young women (UNAIDS 2023). Evidence suggests that HCPs fail to provide HIV services because they lack adequate knowledge and skills to approach patients and caregivers (Mutambo & Hlongwana 2019). This raises concerns about the type of health education being provided by HCPs at the facility and community level, including schools.

The HCPs who participated in the study described treatment failure as the inability of an individual living with HIV to effectively suppress their viral load while adhering to medication. They specifically associated this description with the outcome of viral load levels, focussing on the connection between treatment failure and the inability to achieve VLS. This result is associated with the virologic failure definition and not treatment failure (WHO 2019b). However, treatment failure is defined as the result of three phases: initially virologic failure, followed by immunological failure, and lastly by clinical failure (WHO 2019b). The description of these three phases was attempted by only one of the 29 participants. This is in line with previous studies which demonstrated that HCPs in sub-Saharan Africa lack the skills, knowledge and tools to permit them to provide comprehensive HIV services specifically for children and adolescents (Mutambo & Hlongwana 2019).

This study confirmed that the VLS rate is still low at a facility level, which affects Namibia’s performance regarding the 95-95-95 UNAIDS target and their quest to end AIDS by 2030 (Namibia Strategic Framework for HIV/AIDS 2022; UNAIDS 2022). Most of the HCPs confirmed that the VLS rate in their respective facilities was still below 95% and that more efforts were needed to address this issue in this age group. Few HCPs reported that their facility’s VLS rate was above 95% and good enough to end AIDS by 2030, while others ignored their facility’s VLS status and were not aware of the UNAIDS fast-track target to end AIDS by 2030. The lack of knowledge about the facility’s VLS rate is problematic as the WHO strongly recommends that national programmes and policymakers improve access to viral load testing, motivate people living with HIV to reach and sustain viral suppression and improve the reporting systems which will make health facilities aware of the suppression status and achieve the UNAIDS fast-track target (WHO 2023b).

Healthcare providers’ knowledge about viral load monitoring and VLS is crucial not only for providing high-quality care to people living with HIV but also for preventing new HIV infections in the community and contributing to the global fight against HIV and AIDS (Kubheka, Archary & Naidu 2020). This knowledge empowers HCPs to make informed decisions on the management of individuals living with HIV with or without HVLs. Furthermore, the knowledge empowers and supports HCPs to educate patients and their caregivers on the importance of adherence, ART medication and VLS (Thinn et al. 2019). This confirms Mlambo et al.’s assertion (2021) that lifelong learning is key to improving knowledge, clinical skills and competence. Lastly, HCPs with good knowledge of the HIV cascade should advocate for effective continued education and training of other HCPs at the facility level, including reviewing policies, guidelines, and resources in line with current advancements and developments to combat HIV and AIDS. The VLS rate is key in HIV programme monitoring and should be understood by all HCPs, especially those managing people living with HIV (Brijkumar et al. 2020).

### Current practices in managing older adolescents living with HIV and younger adults living with HIV with high viral loads

The HCPs highlighted key issues in the current management of OALHIV and YALHIV which included the implementation of differentiated service delivery models, psychosocial support, community involvement, the multidisciplinary team or interprofessional approach, enhanced adherence counselling and support from implementing partners. Healthcare providers listed the viraemia clinic day and the DAART as the differentiated service delivery models implemented to address the issues of adherence and improve the VLS, but these initiatives need to be strengthened to produce the intended results by all HCPs. The viraemia clinic day enables all OALHIV and YALHIV with HVLs to be seen on a specific date at the clinic by a multidisciplinary team, and this can be done on days and at times convenient to this age group through their involvement. This is in line with a study by Hanners, Benitez-Burke and Badowski (2022) who found that management of low-level viraemia in people living with HIV still confuses HCPs and that clear guidelines are required to provide direction to the multidisciplinary team.

The core function of multidisciplinary teams is to bring together a group of HCPs from different fields to conduct a root cause analysis to determine patients’ treatment plans. The study conducted on the social determinants of health interventions that improve adherence affirms that multidisciplinary teams play an integral role in ensuring that HIV treatment is successful, and coordinated care from the team improves the rates of ART adherence and other health outcomes (Muzungu 2022). Contrary to phase 2 of this study, the majority of OALHIV and YALHIV expressed dissatisfaction and did not appreciate the multidisciplinary team approach, as this model made them feel uncomfortable and hesitant to open up. This suggests that patient involvement and engagement in care are key in choosing care approaches or models (Clavel et al. 2021; Vahdat et al. 2014).

Enhanced adherence counselling has been standardised through the 2021 Namibian ART guidelines, with more guidance to address the adherence of individuals living with HIV including OALHIV and YALHIV (Ministry of Health and Social Services 2021). In this study, participants listed enhanced adherence counselling as a separate intervention or together with others such as multidisciplinary teams, implementing partner support, DAART and many others, and raised concerns about starting vertical programmes. In a study on the standardised enhanced adherence counselling for improved HIV viral suppression among children and adolescents in Homa Bay and Turkana Counties, Kenya, the proportion of adolescents who suppressed their viral load following enhanced adherence counselling increased by 20%. However, viral load after three of these sessions following standard enhanced adherence counselling implementation was still suboptimal compared to what has been reported in other studies (Masaba et al. 2022).

In another study on enhanced adherence counselling, support groups and VLS among HIV-positive adolescents in a tertiary healthcare facility in Cameroon, the VLS rates were found to be good after completion of the enhanced adherence counselling sessions and participation in support group enrolment for adolescents with HVLs (Agbornkwai et al 2022). Although HIV and TB overlap in several areas, there is an important difference in the management of the two diseases on how directly observed therapy remains successful for TB compared to DAART. Unlike TB, the treatment for HIV infection is administered daily and is lifelong, which made DAART effective in the short run. Furthermore, there are concerns about the feasibility of applying directly observed therapy to lifelong treatment and its acceptability given confidentiality concerns and HIV-related stigma (Uthman et al. 2018).

Psychosocial support plays a major role in the adherence to ART medication among OALHIV and YALHIV. This study summarises the psychosocial support through teen clubs and peer support from NATS as the current management package to address adherence among OALHIV and YALHIV. Okonji et al. (2020) assert that ART adherence and retention in care among young people living with HIV can be sustained by support groups, family-centred services, individual counselling and treatment supporters. In addition, the philosophy of Ubuntu can serve as a valuable approach to address conflicting public health issues and foster an environment where individuals feel comfortable disclosing their HIV status without fear of stigma and discrimination from the community.

The study on the psychosocial support programmes that improve adherence and health systems experiences for adolescents on ART in South Africa demonstrates that adolescents who attended the support group sessions expressed the feeling of not being alone and, as such, were motivated to take their medication regularly (Okonji et al. 2022) This age group is prone to mental health and substance use disorders, which can affect their general health and adherence to antiretroviral drugs. Retention in care therefore requires the integration of mental intervention in HIV management (WHO 2020, 2022).

The community can become involved in the management of OALHIV and YALHIV through the guardians’ support and school involvement, particularly OALHIV and YALHIV in boarding schools. Social support, especially from family members, has been shown to influence treatment adherence. This support can be categorised into emotional, instrumental and appraisal support. Each kind of support provides unique resources to mitigate stressors (Nabunya, Samuel & Ssewamala 2023). Similar to family support, OALHIV and YALHIV in some boarding schools have benefited from the hostel matron or Life Skills teacher while taking their daily dose or when it comes to clinic attendance. This is in line with a study conducted by Kihumuro et al. (2021) in Uganda which revealed that adolescents living with HIV in boarding school face similar challenges to those outside a boarding school setting, namely adherence to medication, stigma and unwillingness to disclose their HIV status. Ubuntu philosophy and its framework were successfully applied during the COVID-19 pandemic to deal with community stigma and discrimination that made most people not to disclose their status and can continue to be effective in efforts to end the HIV epidemic in sub-Saharan Africa (Chigangaidze, Matanga & Katsuro 2022; Jecker, Atuire & Kenworthy 2022).

In addition to the foregoing management package, the implementing partners provide support at the community level and closely follow OALHIV and YALHIV. This support includes tracing the lost to follow up on them, providing psychosocial support and counselling to those with HVLs and providing incentives such as food packages or initiating and supporting OALHIV and YALHIV in income-generating activities. Project HOPE, DREAMS and TCE/Development Aid from People to People were the most listed implementing partners supporting OALHIV and YALHIV. These provide either local or international support through the United States President’s Emergency Plan for AIDS Relief. Mantell et al. (2022) in South Africa emphasise that strengthening and expanding community health work programmes is a great investment but the necessary resources, training and support need to be provided.

The management of OALHIV and YALHIV with HVLs involves a comprehensive approach aimed at achieving VLS, preserving health and preventing further HIV transmission in the community. The WHO’s new consolidated HIV guidelines for prevention, treatment, service delivery and monitoring (2021) support this as a recommendation for a public health approach. It is important to note that the Namibian ART guidelines may change. Therefore, HCPs must receive the latest updates through continuous in-service training, particularly on the management of these age groups. Healthcare providers should take into consideration the unique circumstances of OALHIV and YALHIV and should collaborate with them and their caregivers to achieve a durable VLS. Apart from providing a comprehensive approach, this should be targeted by providing practical, adolescent-friendly health services for adolescents living with HIV to meet their unique needs (WHO 2019a). According to Muhammad-Lawal et al. (2023), Ubuntu can be a driving force or instrument to promote comprehensive and holistic nursing care within the African context.

## Conclusion

This research article provides valuable insights into the knowledge and experiences of HCPs in managing OALHIV and YALHIV. The findings reveal inadequate knowledge among HCPs regarding treatment failure and viral load monitoring; these aspects are key for monitoring the effectiveness of ART and HIV programmes with regard to the last 95 of the UNAIDS target, including achieving the vision of ending new HIV infections by 2030. The findings underscore the need for tailored interventions, HIV programme and support systems to address the unique challenges faced by this population. Healthcare providers’ knowledge needs to be enhanced through continuous in-service training, education, and professional development to improve differentiated service delivery, multidisciplinary teams, community and peer-led approaches within the spirit of Ubuntu. The overall quality of care and health outcomes for older adolescents and younger adults living with HIV can then be improved significantly.
